# Assessment of mesenchymal stem cells for the treatment of spinal cord injury: a systematic review and network meta-analysis

**DOI:** 10.3389/fncel.2025.1532219

**Published:** 2025-04-16

**Authors:** Runfang Wang, Yiding Wang, Fangning Yan, Jinqing Sun, Tianyu Zhang

**Affiliations:** ^1^Department of Medicine, Shandong Xiandai University, Jinan, China; ^2^Tianjin University of Traditional Chinese Medicine, Tianjin, China

**Keywords:** spinal cord injury, mesenchymal stem cells, intrathecal injection, cell transplantation, network meta-analysis

## Abstract

**Objective:**

This study aims to explore the clinical efficacy of mesenchymal stem cell (MSC) transplantation in the treatment of patients with spinal cord injury (SCI) through a network meta-analysis and to discuss the optimal transplantation strategy for treatment.

**Methods:**

We conducted a computer search of clinical randomized controlled studies on MSC treatment for SCI in databases including PubMed, Web of Science, Cochrane Library, Embase, China National Knowledge Infrastructure (CNKI), Chinese Science and Technology Journal Database (VIP), Wanfang Database, and Chinese Biomedical Literature Service System (SinoMed) up to March 2024. Two researchers independently completed literature screening and data extraction according to the inclusion and exclusion criteria and used RevMan 5.4 software to assess the quality of the included studies. Network meta-analysis was performed using Stata 16.0 software.

**Results:**

A total of 18 studies were included in the analysis. The results showed that MSCs significantly improved motor, sensory, and activities of daily living activities after SCI. Network meta-analysis indicated that umbilical cord mesenchymal stem cells (UCMSCs) were the most effective cell source, and intrathecal injection (IT) was the optimal transplantation method.

**Conclusion:**

The study suggests that the current use of UCMSCs for IT transplantation may be the best transplantation strategy for improving functional impairment after SCI. Further high-quality studies are still needed to validate the results of this study and to ensure the reliability of the results.

**Systematic review registration:**

https://www.crd.york.ac.uk/prospero/, identifier [CRD42023466102].

## 1 Background

Spinal cord injury (SCI) is a severe, debilitating traumatic disease of the central nervous system (Yao et al., 2023). SCI is mainly caused by traffic accidents, falls, violence, etc. (Wu et al., 2021). SCI typically results in varying degrees of sensory, motor, and autonomic nervous system dysfunction below the level of injury (Benedetti et al., 2022). The pathogenesis of SCI includes two types: primary and secondary injury. Primary injury mainly leads to damage to the neuronal and glial cell membranes and disruption of the microvascular system, resulting in neuronal death, vascular rupture, and damage to the blood–spinal cord barrier (Stenudd et al., 2015; Taylor et al., 2022). Secondary injury is divided into three stages: acute, subacute, and chronic. The acute phase is within 48 h after injury and is mainly characterized by vascular rupture. The subacute phase involves pathological changes such as neuronal apoptosis and axonal demyelination. If the injury progresses into the chronic phase, it can lead to the formation of cavities and axonal necrosis (Zhang et al., 2021; Liao et al., 2021). SCI can also trigger secondary diseases, including autonomic nervous system dysfunction, cardiovascular diseases, cognitive disorders, and chronic pain (Arora and Craig, 2024). With ongoing research into the pathomechanisms of SCI, treatment methods for SCI have also made corresponding progress, including surgical treatment, drug therapy, cell therapy, hypothermia therapy, hyperbaric oxygen therapy, and rehabilitation therapy. SCI can cause varying degrees of motor and sensory function loss, leading to high treatment and rehabilitation costs and a high rate of disability, imposing a heavy burden on patients and society (Meacham et al., 2022). However, there is currently no clear and effective treatment method, making the exploration of new intervention strategies crucial. Finding effective treatments for SCI has become an urgent problem that needs to be addressed.

Mesenchymal stem cells (MSCs), commonly derived from bone marrow, umbilical cord, adipose tissue, and gingiva, are progenitor cells with the ability for self-renewal, multi-directional differentiation, and immunomodulation (Attia and Mashal, 2021; Tang et al., 2021). MSC transplantation is beneficial for the treatment of SCI, with cellular and molecular mechanisms including the secretion of bioactive molecules that promote neuroprotection, prevent further vascular damage, promote angiogenesis, immunomodulation, axonal regeneration, formation of neuronal relays, and promotion of myelin regeneration (Assinck et al., 2017). A study by Liu et al. (2022) found that after modeling SCI in rats, the levels of pro-inflammatory cytokines, such as IL-1β, IFN-*γ*, IL-6, and TNF-*α* in the serum were elevated. However, after intrathecal (IT) injection of human umbilical cord mesenchymal stem cells (UCMSCs), there was a significant downward trend in IFN-γ, IL-6, and TNF-α levels at 3- and 7-days post-treatment. Moreover, the transplantation of human umbilical cord MSCs into a rat SCI animal model was found to secrete neurotrophic factors, maintaining cell viability in a high oxidative stress microenvironment, leading to effective recovery of the injured spinal cord (Xie et al., 2022). Scholars have also found that transplantation of UCMSCs combined with ultra short-wave therapy can alleviate the inflammatory microenvironment of SCI rats through the NUR77/NF-κB signaling pathway and improve their motor function (Wang et al., 2021). Preclinical animal studies have confirmed the significant potential of MSC transplantation for repairing SCI, but clinical translation has not been smooth (Gao et al., 2020). The optimal clinical transplantation route, dosage, time window, and the mechanisms of MSC transplantation for spinal cord repair, clinical safety, and efficacy still require further exploration.

MSC transplantation is currently a hot topic in the treatment of SCI. Therefore, this study uses network meta-analysis to comprehensively evaluate the intervention effects of different MSC transplantation strategies on motor function, sensory function, and activities of daily living in patients with SCI to determine the best transplantation strategy for clinical decision-making.

## 2 Materials and methods

This systematic review and meta-analysis followed the Preferred Reporting Items for Systematic Reviews and Meta-Analyses (PRISMA) guidelines and was previously registered in the National Institute for Health Research (NIHR) PROSPERO protocol database (International prospective register of systematic reviews, ID CRD 42023466102).

### 2.1 Search strategies

We searched both the Chinese and English databases separately. The English databases included PubMed, Cochrane Library, Web of Science, and Embase. The Chinese databases included China National Knowledge Infrastructure (CNKI), VIP Database for Chinese Technical Periodicals, Wanfang Database, and the China Biomedical Literature Service System (SinoMed). We searched for randomized controlled trials on the treatment of SCI with MSCs in these databases. The time range of the search was from the inception of the database to 5 March 2024.

### 2.2 Study selection criteria

#### 2.2.1 Inclusion criteria

1. Randomized controlled trial of MSC transplantation for SCI in patients.
2. The experimental group was treated with MSCs on top of the control group.
3. The outcome indicator was the American Spinal Injury Association (ASIA), Barthel Index (BI), Modified Barthel Index (MBI), Function Independent Measure (FIM), or Spinal Cord Independence Measure III (SCIM-III).

#### 2.2.2 Exclusion criteria

1. Conference papers or duplicate published studies.
2. Study data could not be extracted.
3. Non-randomized controlled studies.
4. The low-quality score of the literature.

### 2.3 Data extraction

Two researchers used EndNote 20 and manual methods to exclude duplicate studies and retrieved and independently screened articles based on inclusion and exclusion criteria. When there were disagreements, they resolved them through group discussion. One researcher extracted the following information for each included study: first author, publication year, sample size, gender, age, injury level, injury severity, duration of disease, MSC source, MSC transplantation method, MSC dose, final assessment time, and outcome indicators. For studies that assessed outcome indicators at multiple time points, we chose to extract the data from the final assessment for analysis.

There may be differences in pre-treatment ASIA and ADL scores in studies between different groups of patients. The difference in change before and after the intervention and the standard deviation (SD) of the difference in change were calculated for each group according to the formula in the Cochrane Handbook for Systematic Reviews of Interventions. The value of the correlation coefficient (Corr) was 0.5.


$$Mean_{\mathrm{change}}=Mean_{\mathrm{final}}–Mean_{\mathrm{baseline}}\text{.}$$

$$SD\;\mathrm{change}=\sqrt{SD_{\mathrm{baseline}}^{2}+SD_{\mathrm{final}}^{2}-\left(2\times \mathrm{Corr}\times SD_{\mathrm{baseline}}\times SD_{\mathrm{final}}\right)\text{.}}$$

### 2.4 Quality assessment

The risk of literature bias was assessed independently by two investigators using RevMan 5.4 software. Studies were assessed according to the Cochrane Handbook for Systematic Reviews of Interventions. The evaluation included: (a) random sequence generation, (b) allocation concealment, (c) blinding of participants and personnel, (d) blinded assessment of study results, (e) completeness of outcome data, (f) selective reporting of study results, and (g) other biases. Low risk of bias, unclear, and high risk of bias were assessed for each entry. Any disagreement in the above quality assessment process was resolved through group discussion.

### 2.5 Statistical analysis

This study performed the standard meta-analysis and network meta-analysis using Stata 16.0 software.

#### 2.5.1 Standard meta-analysis

Conduct statistical analysis using Stata 16.0 software: Calculate the odds ratio (OR) with statistics and compute the 95% confidence interval (95% CI) for dichotomous variables. Standardized mean difference (SMD) combining statistics were used for continuous variables and 95% CI were calculated. A difference is considered statistically significant if *p* < 0.05. To determine the magnitude of heterogeneity, the Cochrane’s Q test and I^2^ value were used. When *I^2^* ≤ 50%, *p* ≥ 0.1, it suggests low heterogeneity, and a fixed-effects model is adopted; when *I^2^* > 50%, *p* < 0.1, it indicates high heterogeneity, and a random-effects model is used.

#### 2.5.2 Network meta-analysis

Drawing a network relationship compares different mesenchymal stem cell transplantation schemes. Each node in the figure represents a different transplantation scheme, the size of the node represents the sample size using that training scheme, and the thickness of the lines connecting the nodes represents the number of studies included. First, the consistency test is carried out. When there is a closed-loop network, the consistency test is carried out by the node segmentation method. *p* > 0.05 shows that the consistency is good, and the consistency model is used for analysis; When there is no closed loop, the consistency model is directly used for analysis. The effectiveness of different training schemes was compared using the cumulative probability graph for area under the curve (SUCRA). The higher the SUCRA value, the better the effect of the mesenchymal stem cell transplantation scheme. Finally, a funnel plot was drawn to assess the presence of small sample effects and publication bias.

## 3 Results

### 3.1 Study selection

**Figure 1:**
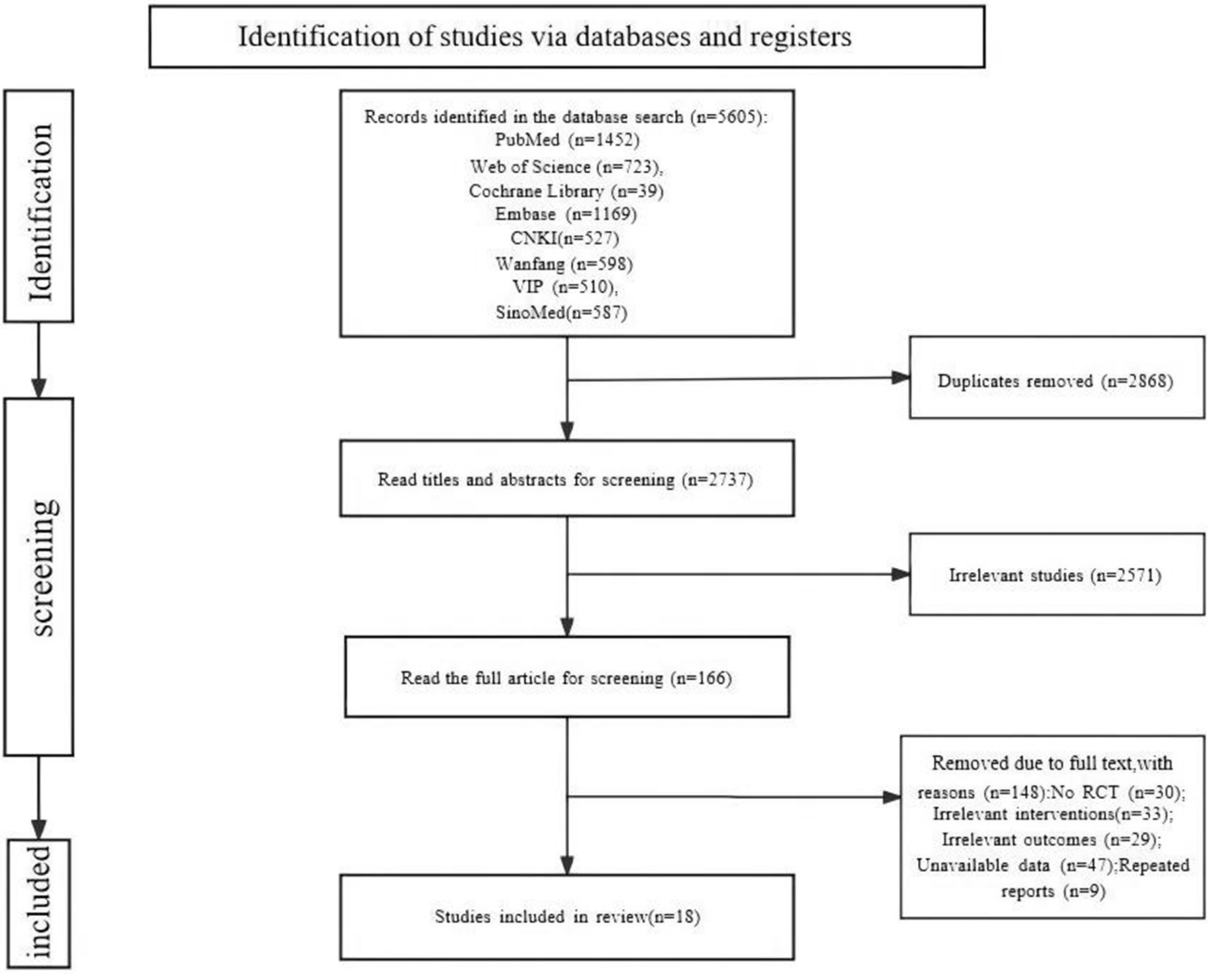
Flow diagram study selection.

A total of 5,605 relevant studies were retrieved. A total of 2,868 duplicate studies were removed. A total of 2,571 studies were removed by reading the titles and abstracts. The full text was read carefully, and 148 studies were removed. Finally, 18 studies (Xie et al., 2007; Abdelaziz et al., 2010; Kishk et al., 2010; Gou et al., 2012; Xiao et al., 2012; Zhang et al., 2012; Dai et al., 2013; Cheng et al., 2014; El-Kheir et al., 2014; Guo et al., 2014; Xiao et al., 2014; Zhang T. et al., 2015; Zhang Z. et al., 2015; Tang et al., 2016; Zhang et al., 2019; Deng et al., 2020; Song et al., 2020; Saini et al., 2022) were included, including 8 (Abdelaziz et al., 2010; Kishk et al., 2010; Dai et al., 2013; Cheng et al., 2014; El-Kheir et al., 2014; Deng et al., 2020; Song et al., 2020; Saini et al., 2022) in English and 10 (Xie et al., 2007; Gou et al., 2012; Xiao et al., 2012; Zhang et al., 2012; Guo et al., 2014; Xiao et al., 2014; Zhang T. et al., 2015; Zhang Z. et al., 2015; Tang et al., 2016; Zhang et al., 2019) in Chinese (Figure 1).

### 3.2 Study characteristics

A total of 950 SCI patients were included in 18 studies, 531 in the MSC group and 419 in the control group. Sources of MSCs included UCMSCs (6 studies) and BMSCs (12 studies). Transplantation routes included intrathecal injection (IT, 7 studies), intralesional injection (IL, 7 studies), and intravenous injection (IV, 2 studies). One study performed a direct comparison between IT and IV. In addition, two studies used IT or/and IV for MSC transplantation. One study did not report the specific transplantation route. Patients in most of the included studies were in the chronic phase of SCI. The essential characteristics of the included studies are shown in Table 1.

**Table 1:** Included studies of MSC transplantation for SCI.

No.	Author	Sample (E/C)	Gender (M/F)	Age (years)	Injury level (C/T/L)	ASIA (A/B/C/D)	Duration (hour/day/month)	Stem cell transplantation			Final evaluation time	Outcomes
								Source	Way	Dose		
1	Xie et al. (2007)	11/13	9/2;10/3	(18–49)/ (21–53)	2/4/5;3/4/6	8/0/1/2;9/1/2/1	(1–10)/ (1–12) mo	Auto, BMSCs	IT or IV	IT: (4.08–10.2) × 10^7^ IV: (4.87–8.8) × 10^7^	3 months	①②③⑤
2	Abdelaziz et al. (2010)	20/10	25/5	6–52;6–64	0/20/0; −	10/5/5/0; −	27.15 mo; −	Auto, BMSCs	IL	2 × 10^6^	12 months	③
3	Kishk et al. (2010)	43/20	36/7;15/5	(31.7 ± 10.4)/ (33.8 ± 11.8)	6/37/0;2/18/0	-	(3.6 ± 2.5)/ (3.7 ± 2.1) y	Auto, BMSCs	IT	(5–10) × 10^6^	12 months	③
4	Gou et al. (2012)	12/12	11/1;10/2	29/31	-	-	2.3/2.5 mo	Allo, UCMSCs	1 time IV, 3 times IT	(2–5) × 10^7^	5 months	①②⑤
5	Xiao et al. (2012)	38/32/26	25/13; 21/11;17/9	(42.3 ± 10.2)/ (41.5 ± 10.7)/ (41.2 ± 10.6)	7/15/16; 6/12/14;5/9/12	-	(25.2 ± 6.7) d	Auto, BMSCs	IT /IV	-	6 months	①②
6	Zhang et al. (2012)	30/30	50/10	32.5 ± 4.2	12/20/28	26/20/12/2	1–10 mo	Allo, UCMSCs	IV	1 × 10^7^	3 months	①②⑤
7	Dai et al. (2013)	20/20	14/6;14/6	(34.7 ± 8.9)/ (35.1 ± 8)	20/0/0; 20/0/0	20/0/0/0; 20/0/0/0;	(51.9 ± 18.3)/ (43.2 ± 15.3) mo	Auto, BMSCs	IL	8 × 10^5^	6 months	①③
8	Cheng et al. (2014)	10/10	-	(35.3 ± 8.23)/ (32.4 ± 7.72)	-	10/0/0/0;10/0/0/0	(21.4 ± 12.96)/ (30.7 ± 19.99) mo	Allo, UCMSCs	IL	2 × 10^7^	6 months	①②④
9	El-kheir et al. (2014)	15/10; 35/10	61/9	16–45	7/8/0;6/4/0; 3/32/0;1/9/0	15/0/0/0;10/0/0/0; 0/35/0;0/10/0	(18.25 ± 5) mo	Auto, BMSCs	IT	2 × 10^6^	18 months	①③
10	Gou et al. (2012)	40/40	30/10;33/7	(37.25 ± 1.96)/ (36.37 ± 1.88)	13/3/24;17/3/20	-	>1mo	Auto, BMSCs	IL	(2–4) × 10^2^	7 days	①②④
11	Xiao et al. (2014)	35/29	23/12;19/10	(42.8 ± 10.2)/ (41.4 ± 10.5)	7/12/16;6/10/13	7/12/16;6/10/13	(5.6 ± 2.6)/ (5.3 ± 2.5) h	Auto, BMSCs	IT	(2.5–10) × 10^5^	6 months	①②
12	Zhang T. et al. (2015)	50/50	16/34;15/35	(35.39 ± 1.85)/ (36.4 ± 2.06)	-	-	-	Auto, BMSCs	-	(2–4) × 10^2^	7 days	①②④
13	Zhang Z. et al. (2015)	15/15	11/4;11/4	(35.5 ± 8.3)/ (35.7 ± 8.3)	10/5/0; 10/5/0	9/3/3/0; −	(21.3 ± 5.7)/ (19.7 ± 7.6) mo	Allo, UCMSCs	IL	8 × 10^5^	6 months	①
14	Tang et al. (2016)	30/30	21/9;20/10	(38.2 ± 6.7)/ (37.9 ± 7.2)	0/13/17;0/14/16	8/6/7/9;7/8/8/7	(5.2 ± 1.2)/ (4.9 ± 1.1) d	Auto, BMSCs	IT	1 × 10^8^	(12.4 ± 2.3) mo	①⑤
15	Zhang et al. (2019)	50/50	30/20;28/22	(41.26 ± 9.74)/ (42.89 ± 10.3)	0/42/8;0/44/6	0/16/20/14;0/15/22/13	(6.55 ± 2.43)/ (6.80 ± 2.66) h	Allo, UCMSCs	IT	(3–4) × 10^7^	12 months	①②③⑦
16	Deng et al. (2020)	20/20	5/15;7/13	(33.7 ± 9.03)/ (34.55 ± 10.89)	20/0/0;20/0/0	-	(12.45 ± 5.74)/ (13.1 ± 5.23) d	Allo, UCMSCs	IL	4 × 10^7^	12 months	①②③
17	Song et al. (2020)	18/18	12/6;10/8	(41.2 ± 2.3)/ (41.7 ± 2.1)	9/4/3(C and T:1, T and L:1);10/5/2(C and T:1)	-	-	Auto, BMSCs	IT	1 × 10^7^	12 months	①②⑦
18	Saini et al. (2022)	7/6	-	18–50	-	7/0/0/0;6/0/0/0	21d	Auto, BMSCs	IL	2 × 10^8^	6 months	②③⑦

“-” indicates no relevant data. ① ASIA motor score, ② ASIA sensory score, ③ ASIA grade improvement, ④ BI, ⑤ MBI, ⑥ FIM, and ⑦ SCIM-III. Data were presented as mean ± SD or n. E/C, experiment/control; M/F, male/female; C/T/L, cervical/thoracic/lumbar spinal cord; SD, Sprague–Dawley; Auto, autologous MSCs; Allo, allogeneic MSCs.

### 3.3 Study quality and risk of bias

**Figure 2:**
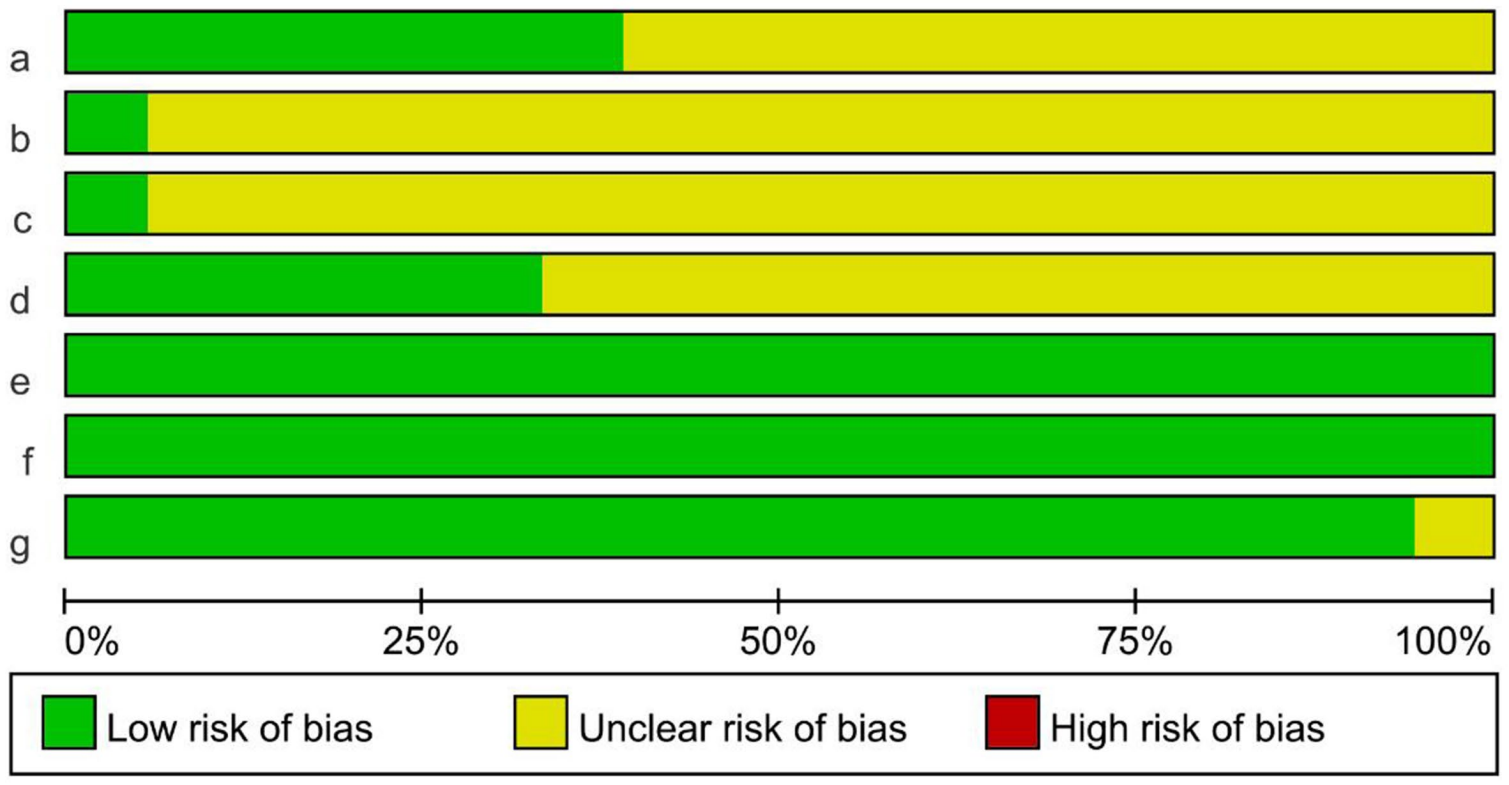
Risk assessment of bias in the included studies.

The Cochrane risk-of-bias tool was used to evaluate the bias risk of the included studies. The evaluation content included six items: random mode, allocation concealment, blind method, incomplete reporting risk, selective reporting risk, and other bias. The corresponding evaluation was made according to the three levels of “low risk,” “unclear,” and “high risk.” The risk of bias evaluation of the included studies is shown in Figure 2. Seven studies mentioned specific random assignment methods. One study used opaque envelopes for allocation concealment. One study was applied for blind participants and personnel. Six studies were blinded to the assessors. All literature was free from missing study data and selective reporting. One study had another bias.

### 3.4 Standard meta-analysis

#### 3.4.1 ASIA motor scores

**Figure 3:**
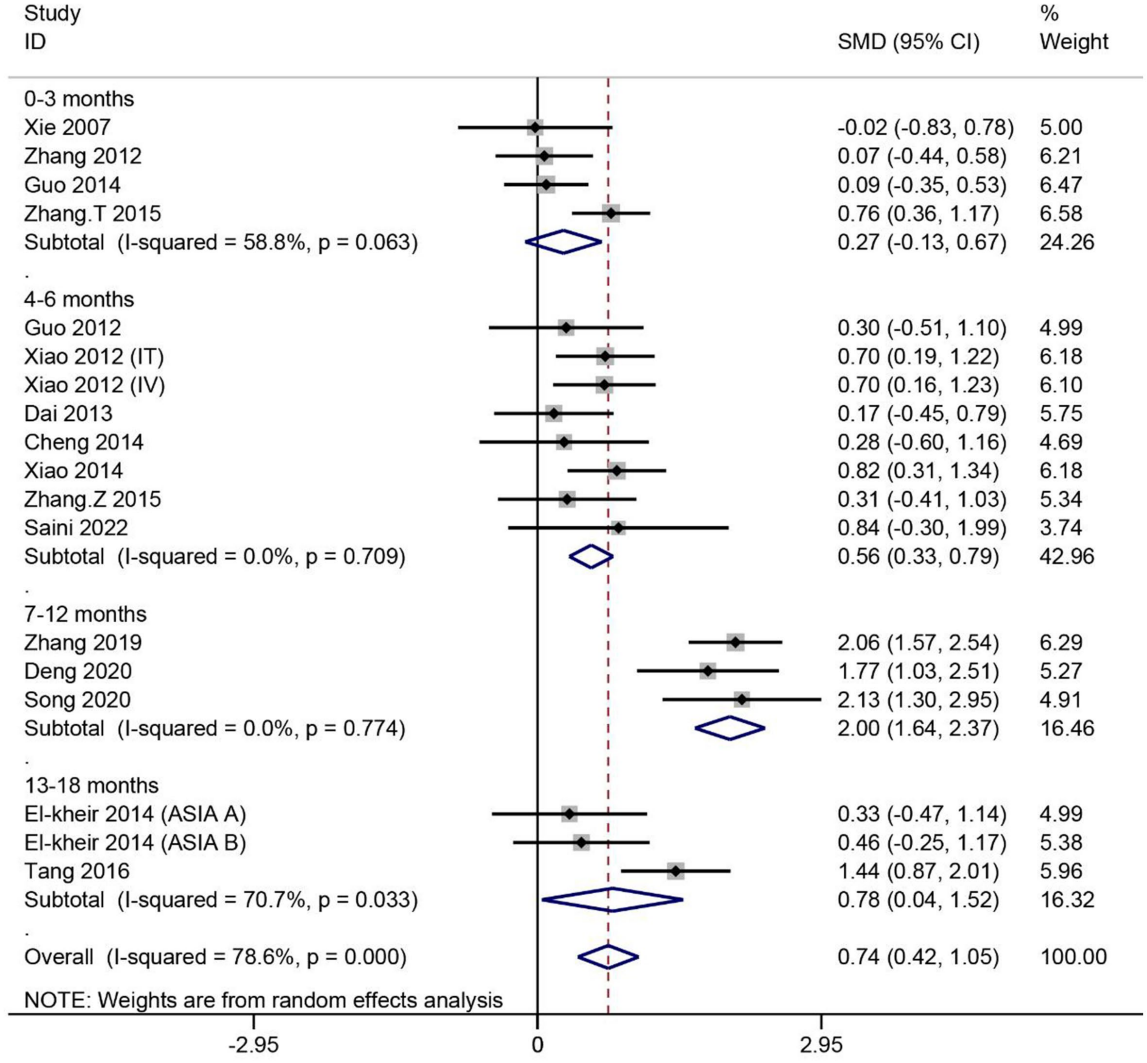
Meta-analysis of ASIA motor scores in SCI patients.

A total of 16 articles reported the effect of MSCs on ASIA motor scores in patients with SCI. Heterogeneity among studies was significant (*I^2^* = 78.6%, *p <* 0.1). Subgroup analysis based on different evaluation times showed high heterogeneity in two of the four subgroups (*I^2^* > 50%, *p* < 0.1). Data were combined using the random effects model. The results showed no significant difference in ASIA motor scores between the MSC group assessed at 0–3 months and the control group (SMD = 0.27, 95% CI -0.13–0.67, *p* > 0.05). For the remaining three subgroups, the ASIA motor scores were significantly higher in the MSC group than in the control group (4–6 months: SMD = 0.56, 95% CI 0.33–0.79, *p* < 0.05; 7–12 months: SMD = 2.00, 95% CI 1.64–2.37, *p* < 0.05; 13–18 months: SMD = 0.78, 95% CI 0.04–1.52, *p* < 0.05) (Figure 3).

#### 3.4.2 ASIA sensory scores

**Figure 4:**
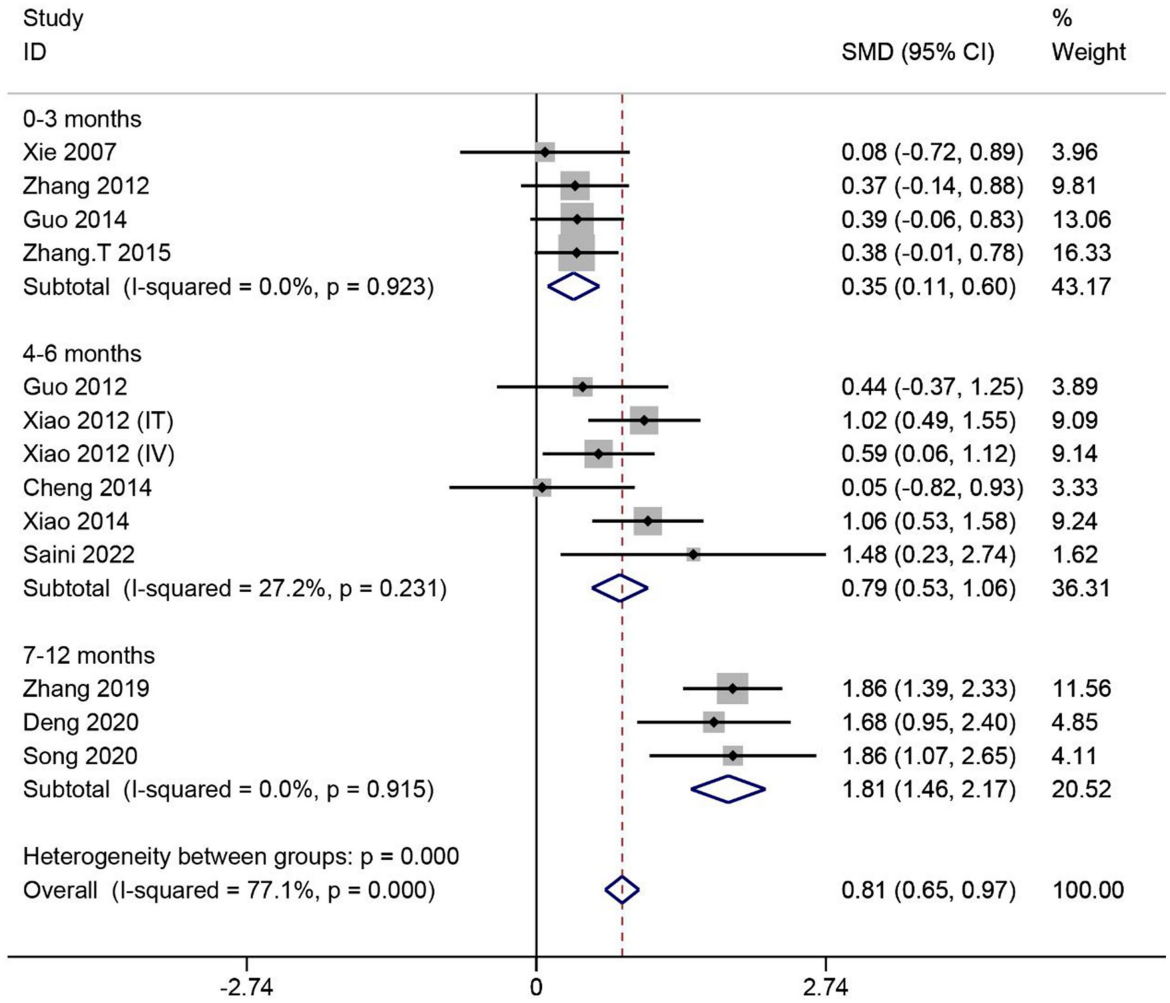
Meta-analysis of ASIA sensory scores in SCI patients.

A total of 12 articles reported the effect of MSCs on ASIA sensory scores in SCI patients. Heterogeneity among studies was significant (*I^2^* = 77.1%, *p* < 0.1). Subgroup analysis based on different evaluation times showed low heterogeneity in three subgroups (*I^2^* < 50%, *p* > 0.1). Data were combined using the fixed effects model. The results showed that the ASIA sensory scores were significantly higher in the MSC group than in the control group in three subgroups (0–3 months: SMD = 0.35, 95% CI 0.11–0.60, *p* < 0.05; 4–6 months: SMD = 0.79, 95% CI 0.53–1.06, *p* < 0.05; 7–12 months: SMD = 1.81, 95% CI 1.46–2.17, *p* < 0.05) (Figure 4).

#### 3.4.3 ASIA grade improvement

**Figure 5:**
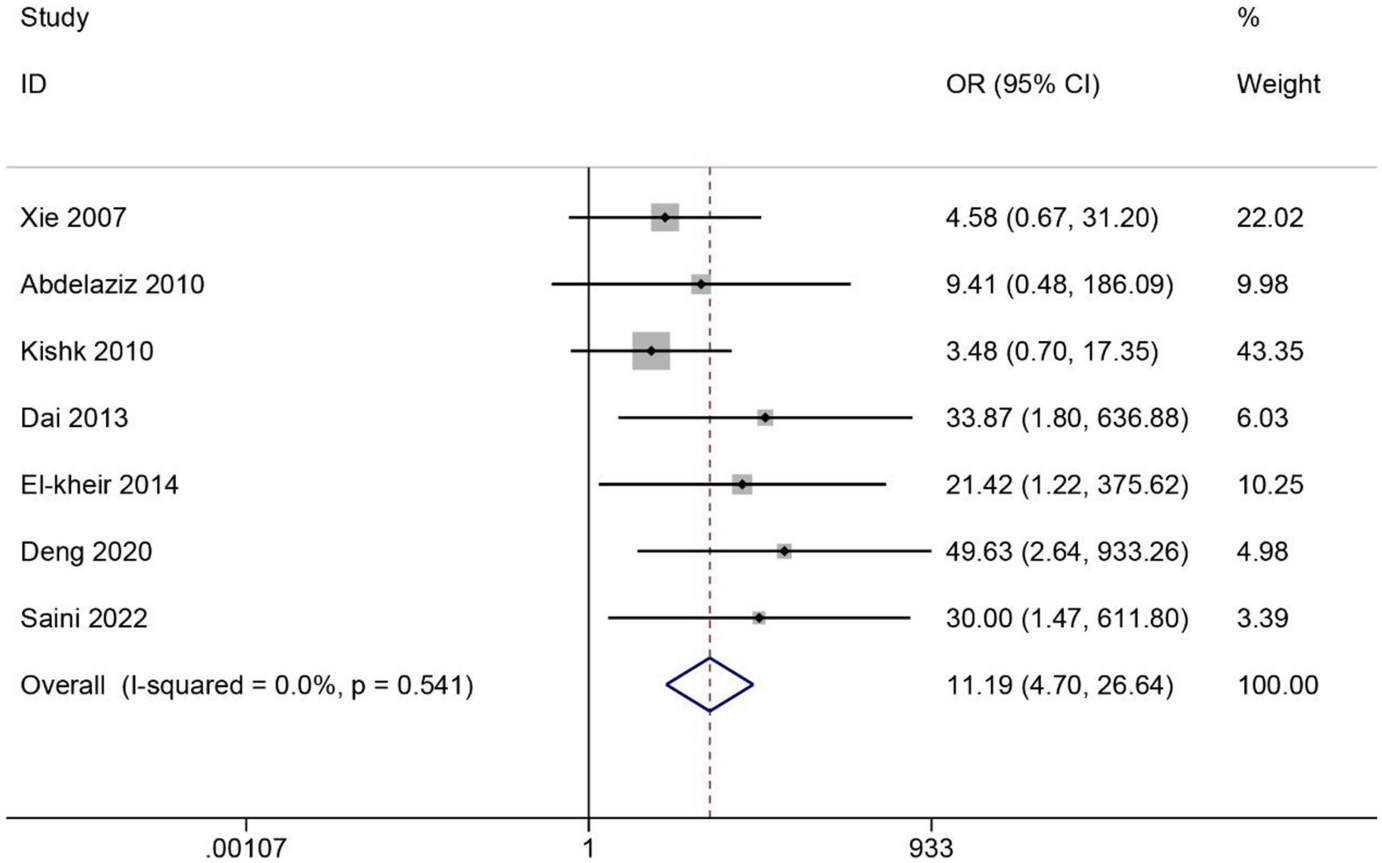
Meta-analysis of ASIA grade improvement in SCI patients.

A total of 8 articles reported the effect of MSCs on the rate of improvement of ASIA grade in SCI patients. Heterogeneity among studies was significant (*I^2^* = 43%, *p* < 0.1) (Supplementary Figure 1). After excluding one article, *I^2^* = 0% and *p* > 0.1. Data were combined using the fixed effects model. The results showed that the rate of improvement in ASIA grade was significantly higher in the MSC group than in the control group (OR = 11.19, 95% CI 4.70–26.64, *p* < 0.05) (Figure 5).

#### 3.4.4 Activities of daily living

**Figure 6:**
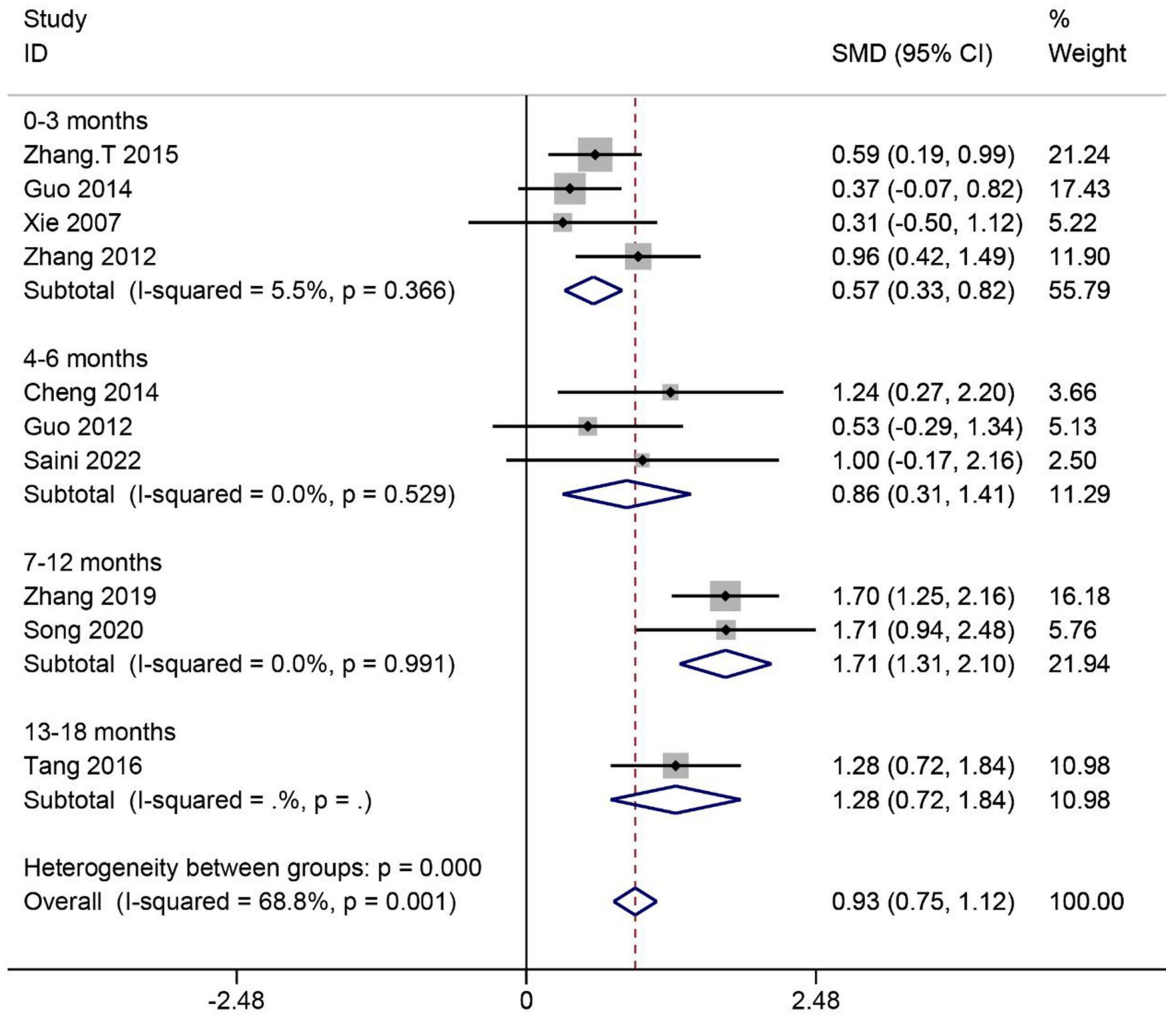
Meta-analysis of ADL scores in SCI patients.

A total of 10 articles reported the effect of MSCs on activities of daily living (ADL) scores in SCI patients. Heterogeneity among studies was significant (*I^2^* = 68.8%, *p* < 0.1). Subgroup analysis based on different evaluation times showed low heterogeneity in four subgroups (*I^2^* < 50%, *p* > 0.1). Data were combined using the fixed effects model. The results showed that the ADL scores were significantly higher in the MSC group than in the control group in four subgroups (0–3 months: SMD = 0.57, 95% CI 0.33–0.82, *p* < 0.05; 4–6 months: SMD = 0.86, 95% CI 0.31–1.41, *p* < 0.05; 7–12 months: SMD = 1.71, 95% CI 1.31–2.10, *p* < 0.05; 13–18 months: SMD = 1.28, 95% CI 0.72–1.84, *p* < 0.05) (Figure 6).

### 3.5 Network meta-analysis

#### 3.5.1 Stem cell transplantation source

**Figure 7:**
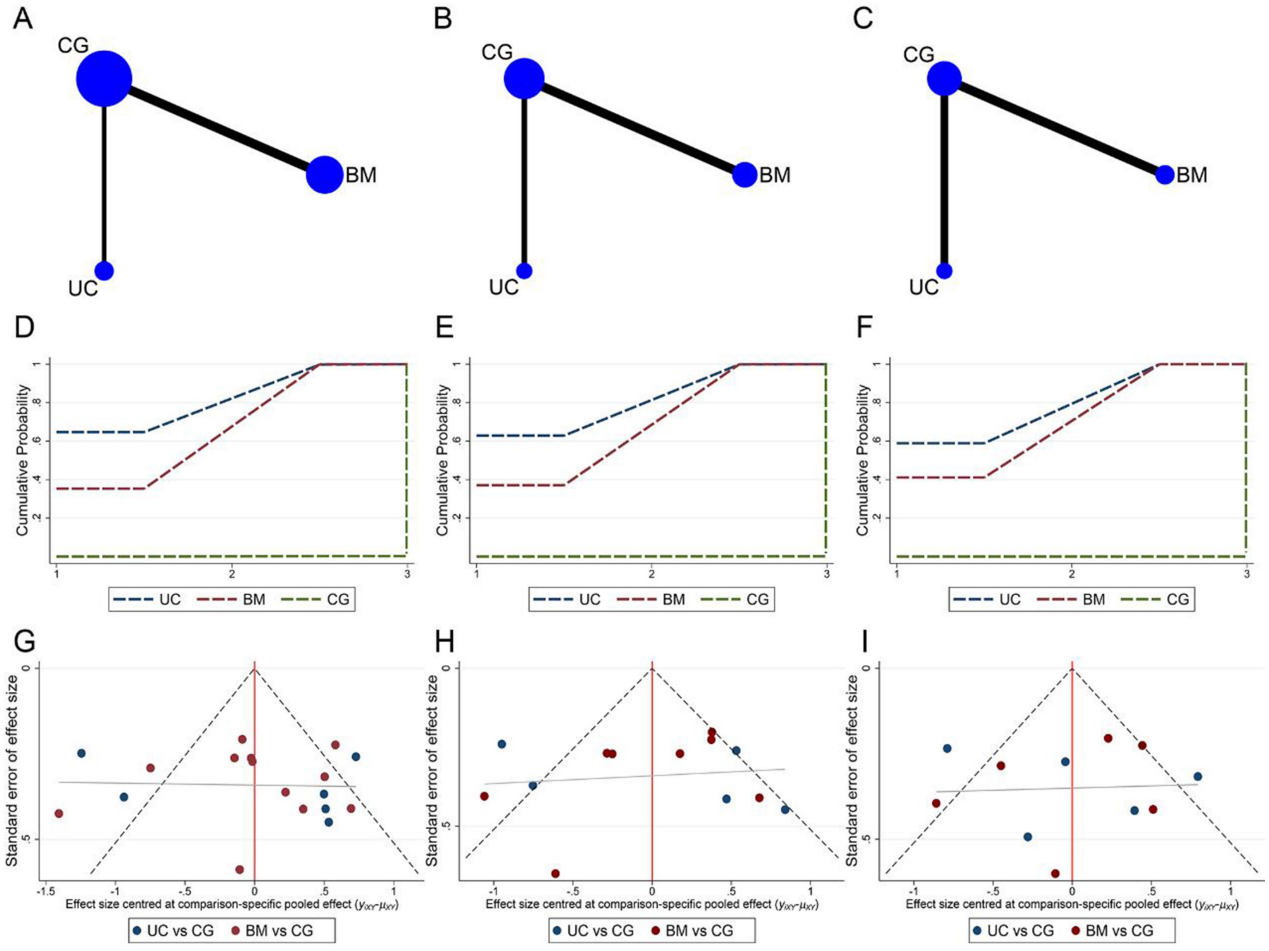
Network meta-analysis of included studies with different MSCs cell sources. **(A)** Network relationship of ASIA motor scores from different cell sources; **(B)** Network relationship of ASIA sensory scores from different cell sources; **(C)** Network relationship of ADL scores from different cell sources; **(D)** SUCRA of ASIA motor scores; **(E)** SUCRA of ASIA sensory scores; **(F)** SUCRA of ADL scores; **(G)** Funnel plot of ASIA motor scores; **(H)** Funnel plot of ASIA sensory scores; **(I)** Funnel plot of ADL scores.

Network meta-analysis of ASIA motor, sensory, and ADL assessment results according to different cell sources. The network relationship diagrams are sequentially shown in Figures 7A–C. Overall, the number of studies with direct comparisons between BMSCs and the control group was higher. No closed loop was formed between the interventions, so no consistency test was required. The SUCRA rankings of the different interventions for outcome indicators were both UCBMSCs > BMSCs > CG (ASIA motor: 82.2% > 67.7% > 0.1%, Figure 7D; ASIA sensory: 81.4% > 68.5% > 0.1%, Figure 7E; ADL: 79.4% > 70.5% > 0.0%, Figure 7F). The imperfectly symmetrical comparison-corrected funnel plots suggest a possible publication bias and a small sample effect in the above results (Figures 7G–I).

#### 3.5.2 Stem cell transplantation way

**Figure 8:**
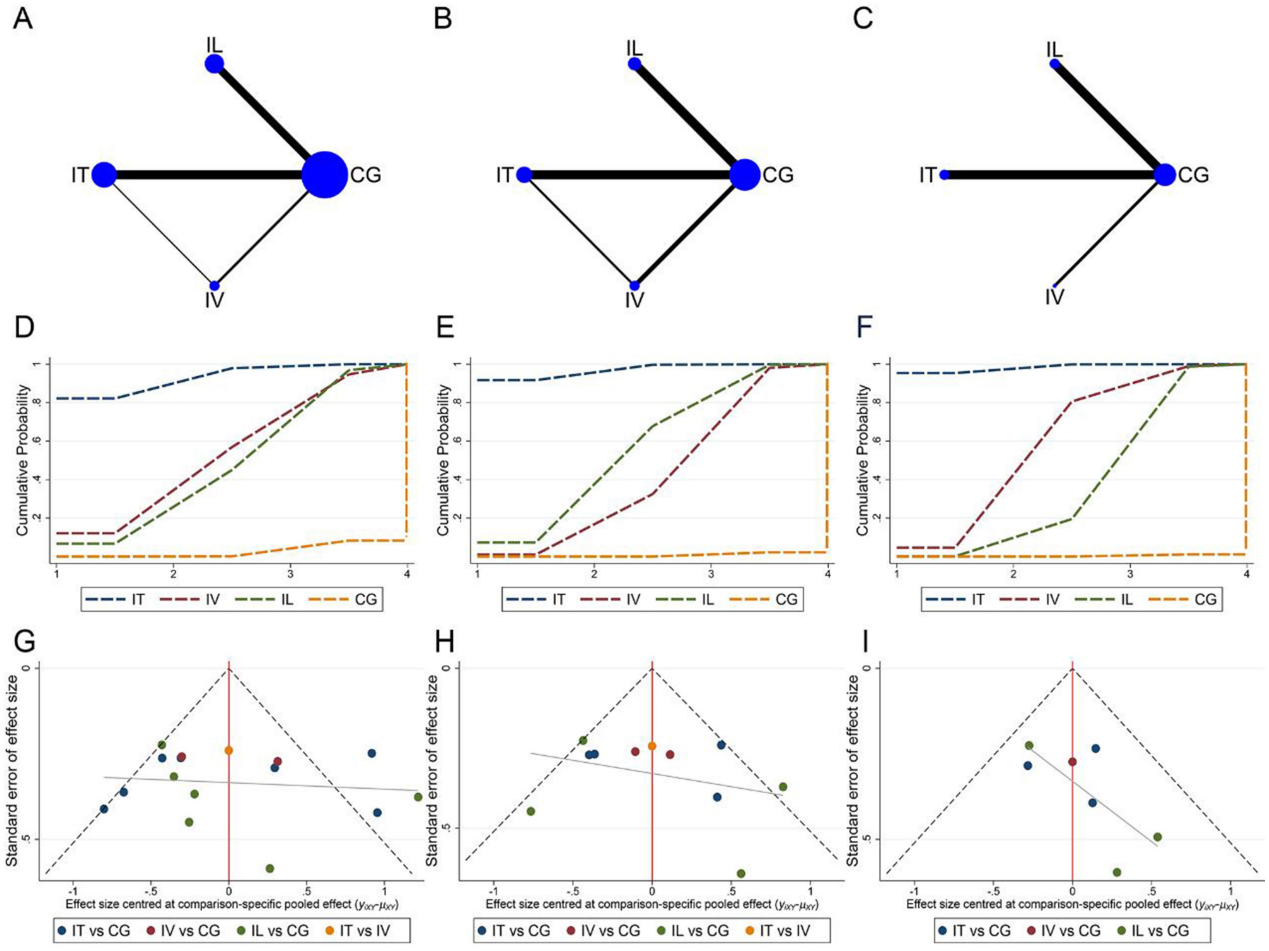
Network meta-analysis of included studies with different MSCs transplantation methods. **(A)** Network relationship of ASIA motor scores for different transplantation methods; **(B)** Network relationship of ASIA sensory scores for different transplantation methods; **(C)** Network relationship of ADL scores for different transplantation methods; **(D)** SUCRA of ASIA motor scores; **(E)** SUCRA of ASIA sensory scores; **(F)** SUCRA of ADL scores; **(G)** Funnel plot of ASIA motor scores; **(H)** Funnel plot of ASIA sensory scores; **(I)** Funnel plot of ADL scores.

Network meta-analysis of the ASIA motor, sensory, and ADL assessment results according to different cell transplantation ways. Network relationship plots are shown sequentially in Figures 8A–C. Overall, there were more studies directly comparing IT and control groups. Local inconsistency tests were performed separately for the closed loops in Figures 8A,B. The results showed *p* > 0.05, so the consistency model was used. The SUCRA rankings for different interventions using ASIA motor scores or ADL scores as outcome indicators were both IT > IV > IL > CG (ASIA motor: 93.1% > 54.5% > 49.6% > 2.8%, Figure 8D; ADL: 98.5% > 61.6% > 39.4% > 0.5%, Figure 8F). While using ASIA sensory score as an outcome indicator, the SUCRA ranking for different interventions was IT > IL > IV > CG (97.1% > 58.3% > 43.9% > 0.7%, Figure 8E). The imperfectly symmetrical comparison-corrected funnel plots suggest a possible publication bias and a small sample effect in the above results (Figures 8G–I).

## 4 Discussion

SCI refers to damage that acts upon the spinal cord and causes temporary or permanent changes in its function. It often results in partial or complete loss of sensation, movement, and visceral perception at the site of injury. With the growth of the world’s population, the number of patients with spinal cord injuries has increased, but the age-standardized prevalence of SCI has not significantly changed (GBD 2016 Traumatic Brain Injury and Spinal Cord Injury Collaborators, 2019). Currently, many clinical trials of stem cell transplantation are in the early stages (Phase I/II), and their effectiveness and safety have been preliminarily confirmed (Yamazaki et al., 2020). This study conducted a standardized meta-analysis of 18 clinical controlled trials and found that MSC transplantation significantly improved motor function, sensory function, and activities of daily living in patients with SCI. We selected the ASIA motor function score, ASIA sensory function score, and ADL score as evaluation indicators and conducted a network meta-analysis to comprehensively compare the effects of different MSC cell sources and transplantation methods. Studies have shown that the heterogeneity of Asia motor and sensor scores subgroup analysis of SCI patients after MSC transplantation is high. This may be related to the small sample size of some included studies, the absence of strict inclusion and exclusion criteria, the absence of standardized interventions, and the absence of clear provisions for the use of a unified assessment scale and assessment time point.

Currently, UCMSCs and bone marrow mesenchymal stem cells (BMSCs) are commonly used in the clinical treatment for spinal cord injuries. When injecting stem cells, factors such as the method of injection, dosage, and timing of injection need to be considered. The main methods of stem cell injection include IT, intralaminar (IL), and intravenous (IV) injections. The results revealed that, from the perspective of the cell source, UCMSC transplantation is the most effective for treating SCI. In terms of transplantation methods, IT injection of stem cells can be directly injected into the injured spinal cord, and compared to other administration routes, it has a longer cell survival time and the best effect. Chen et al. (2021) found through meta-analysis that the IT and transplantation approach can improve the neurological function of SCI patients the most. Lu et al. (2022) found that the therapeutic effect of IT was significantly better than that of IV based on the systematic review and meta-analysis of animal studies.

UCMSCs are primarily found in the Wharton’s jelly of the umbilical cord, umbilical cord blood, and tissues surrounding the umbilical cord blood vessels (Lu et al., 2022). Minimally invasive IT injection into the subarachnoid space is considered the safest and most effective method for delivering UCMSCs (Yang et al., 2020). BMSCs have been discovered for the first time in bone marrow and have the potential to be a breakthrough in treating spinal cord injuries due to their properties of neuroregeneration, neuroprotection, and immunomodulation (Wang et al., 2019). However, immune rejection and the potential for tumor formation limit their application. The therapeutic effect of BMSCs largely depends on the release of soluble paracrine factors, with exosomes (EXO) being essential. BMSC-derived EXO is expected to replace the function of BMSCs (Zhou et al., 2022). Compared to other stem cells, BMSCs can be sourced from autologous transplantation, thus avoiding social and ethical issues.

In summary, intramedullary injection of stem cells can improve the sensory and motor function scores and the ability to perform activities of daily living in patients with SCI, initially proving its effectiveness. However, there are still many issues that require further study.

The limitations of this study are as follows:

1. Among the 18 studies included in this article, some had small sample sizes, and most did not implement blinding or allocation concealment, which may cause bias. These limitations may affect the reliability of the meta-analysis results. It is recommended to increase the sample size and strictly implement blinding and allocation concealment in future research to provide higher quality evidence.
2. Some articles included in the analysis did not explain whether the applied MSCs met the minimum standards proposed by the International Society for Cellular Therapy (ISCT), which had a certain impact on the therapeutic effect and the accuracy of the research results.
3. Although this study ranked the advantages and disadvantages of MSC transplantation treatment for SCI, it cannot currently explain the reasons for their formation. In the future, it is necessary to establish a clinical level and standardized MSC system to facilitate homogeneous basic experiments and clinical research. Further randomized controlled clinical studies with large samples need to be carried out to apply MSCs that meet ISCT standards to verify the optimal transplantation route, dose, and timing of MSCs.

## 5 Conclusion

In summary, by analyzing the sensory and motor function scores of the ASIA scale, the improvement of ASIA grades, and the ADL scores of included SCI patients, the application of IT transplantation of UCMSCs may be the best transplantation strategy for improving functional disabilities after SCI. Future research should further clarify its mechanism of action, focus on the safety and efficacy of MSC transplantation treatment for SCI, and explore more scientific and effective transplantation protocols.

## Data Availability

The original contributions presented in the study are included in the article/Supplementary material, further inquiries can be directed to the corresponding authors.
