# Supplementary figures and images for: Assessment of mesenchymal stem cells for the treatment of spinal cord injury: a systematic review and network meta-analysis
Source: Front Cell Neurosci. 2025 Apr 16;19:1532219. doi: 10.3389/fncel.2025.1532219 (PMC12040839; doi:10.3389/fncel.2025.1532219)

**Supplemental Fig. 1 Meta-analysis of ASIA grade improvement in SCI patients.**

**
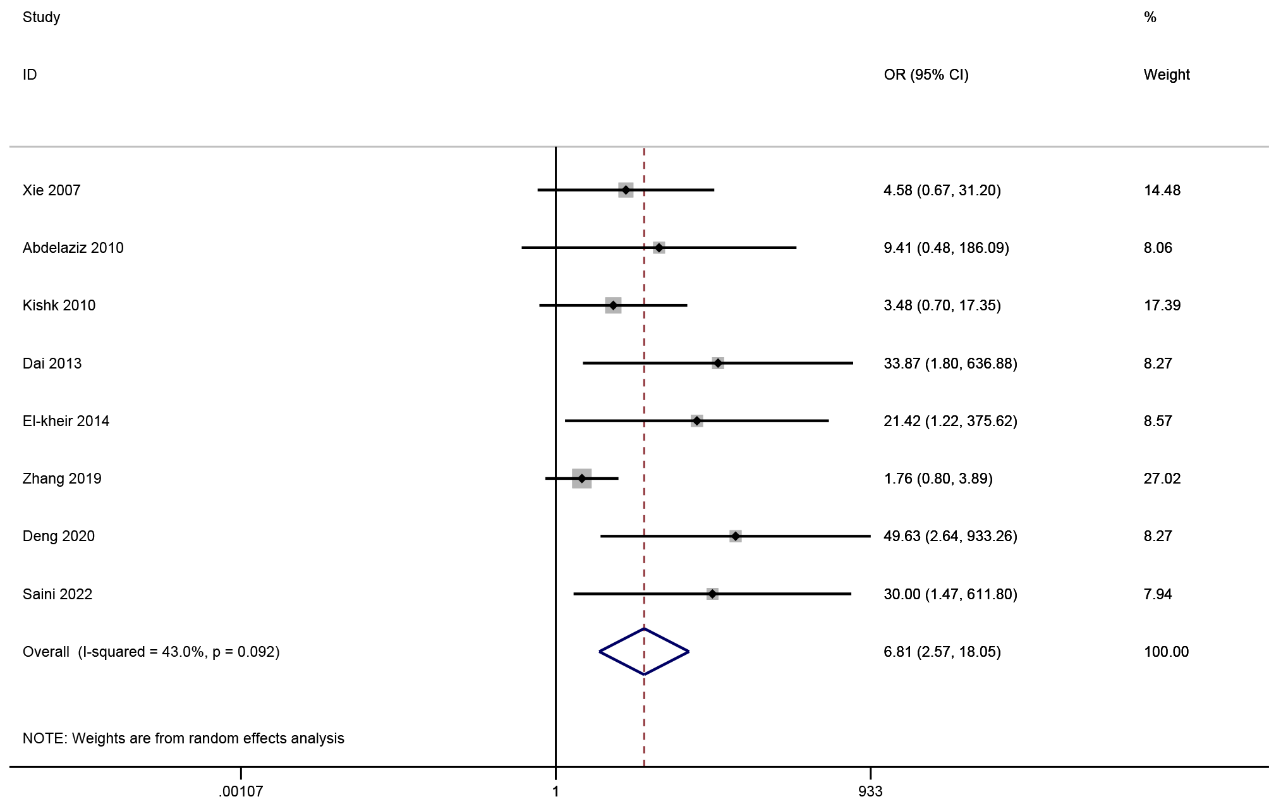
**

Supplement: Supplementary file 1 [file Data_Sheet_1.docx]
